# Ecophysiology and Growth of White Spruce Seedlings from Various Seed Sources along a Climatic Gradient Support the Need for Assisted Migration

**DOI:** 10.3389/fpls.2017.02214

**Published:** 2018-01-08

**Authors:** Guillaume Otis Prud'homme, Mohammed S. Lamhamedi, Lahcen Benomar, André Rainville, Josianne DeBlois, Jean Bousquet, Jean Beaulieu

**Affiliations:** ^1^Centre d'étude de la Forêt, Faculté de Foresterie, de Géographie et de Géomatique, Université Laval, Québec City, QC, Canada; ^2^Direction de la Recherche Forestière, Ministère des Forêts, de la Faune et des Parcs, Québec City, QC, Canada; ^3^Canada Research Chair in Forest Genomics, Université Laval, Québec City, QC, Canada

**Keywords:** assisted migration, white spruce, climate change, local adaptation, acclimation, vector analysis, photosynthesis before bud break, mineral nutrition

## Abstract

With climate change, favorable growing conditions for tree species are shifting northwards and to higher altitudes. Therefore, local populations are becoming less adapted to their environment. Assisted migration is one of the proposed adaptive measures to reduce the vulnerability of natural populations and maintain forest productivity. It consists of moving genetic material to a territory where future climate conditions correspond to those of its current location. Eight white spruce (*Picea glauca* [Moench] Voss) seed sources representing as many seed orchards were planted in 2013 at three forest sites simulating a south-north climatic gradient of 1.7°C in Québec, Canada. The objectives were to (1) evaluate the morpho-physiological responses of the different seed sources and (2) determine the role of genetic adaptation and physiological plasticity on the observed variation in morpho-physiological traits. Various seedling characteristics were measured, notably height growth from nursery to the fourth year on plantation. Other traits such as biomass and carbon allocation, nutritional status, and various photosynthetic traits before bud break, were evaluated during the fourth growing season. No interaction between sites and seed sources was observed for any traits, suggesting similar plasticity between seed sources. There was no change in the rank of seed sources and sites between years for height growth. Moreover, a significant positive correlation was observed between the height from the nursery and that after 4 years in the plantation. Southern seed sources showed the best height growth, while optimum growth was observed at the central site. Juvenile height growth seems to be a good indicator of the juvenile carbon sequestration and could serve as a selection criterion for the best genetics sources for carbon sequestration. Vector analysis showed no nitrogen deficiency 4 years after planting. Neither seed sources nor planting sites had a significant effect on photosynthesis before bud break. The observed results during the establishment phase under different site conditions indicate that southern seed sources may already benefit from assisted migration to cooler climatic conditions further north. While northern seed sources are likely to benefit from anticipated local global warming, they would not match the growth performance of seedlings from southern sources.

## Introduction

The geographic distribution of forest species is influenced by climate (Davis and Shaw, 2001). Over the course of recent millennia, forest tree species have adapted to local conditions and populations often vary genetically along a clinal gradient (Andalo et al., 2005; Aitken and Whitlock, 2013). With the global warming predicted by General Circulation Models (GMCs) and various scenarios envisioned for the boreal zone (GIEC, 2014), we should expect a shift of current climatic niches northwards or to higher altitudes (e.g., Papadopol, 2000; Davis and Shaw, 2001). In response to this pressure, forest tree populations will have to adapt to changing local conditions or migrate to environments matching their present climate. However, the speed of migration of forest species is 10–100 times slower than that predicted for the shift in climatic niches (NRC, 2016), which leads us to anticipate maladaptation, even of minor importance, of local populations to new conditions.

Assisted population migration, which consists of relocating seed sources used for reforestation to habitats to which they are optimally adapted within species range, is currently one of the proposed strategies to mitigate the effect of climate change on forest productivity and to reduce the vulnerability of forested ecosystems (Mueller and Hellmann, 2008; Aubin et al., 2011; Pedlar et al., 2012). Assisted population migration is less risky compared to assisted range expansion and long distance species migration (Pedlar et al., 2012). However, its implementation must be based on solid scientific knowledge to minimize the associated risks. Seed source transfer models have already been developed for many tree species, using growth measurements collected in provenance trials replicated on several sites (Beaulieu et al., 2004; Andalo et al., 2005; Thomson and Parker, 2008; Thomson et al., 2009; O'Neill et al., 2017). These models establish a relationship between tree growth and climatic variables. The first model for white spruce in Québec was developed by Andalo et al. (2005). It was later refined by using estimates of a set of climate variables either obtained from weather station 30-year-normal records or various GMCs and scenarios to predict plantation yield (m^3^/ha) as a function of climate differences between two periods and/or sites (Rainville et al., 2014).

Growth is a characteristic that is strongly influenced by the interaction between seedling genotype and available resources (water, soil fertility, etc.), as well as by environmental stresses (Li et al., 2017). Therefore, it would be advisable to complement growth measurements with the assessment of other variables in order to reduce the risks associated with assisted migration (Isaac-Renton et al., 2014). Evaluating morpho-physiological traits of seed sources used in reforestation programs in response to their growing conditions should contribute to the development of operational recommendations that are not only more exhaustive, but also less risky in terms of optimal transfer distances.

White spruce (*Picea glauca* [Moench] Voss) is one of the most important commercial species in the North American boreal forest (Beaulieu et al., 2009). Its genetic improvement programs are among the most advanced (Mullin et al., 2011). For example, more than 98% of the seedlings produced for reforestation in Québec are grown from improved seed (Lamhamedi and Carles, 2012). It is already known that certain morpho-physiological traits (e.g., traits related to growth or photosynthesis) of white spruce seedlings vary between seed orchards and regions (Carles et al., 2011; Benomar et al., 2015; Villeneuve et al., 2016), families (Carles et al., 2012), and somatic clones (Lamhamedi et al., 2000; Wahid et al., 2012), attesting to local genetic adaptation. Some of these traits may also be highly influenced by site and environmental characteristics (soil properties and fertility, temperature, precipitation, etc.), demonstrating the existence of considerable physiological plasticity (Benomar et al., 2016). However, our previous studies carried out under controlled conditions showed a stability of morphological characteristics in response to the interaction between a simultaneous increase in CO_2_ concentration and temperature (Carles et al., 2015).

Until now, few studies have evaluated morpho-physiological responses of seedlings originating from seed orchards representative of geographically distinct sources several years after planting on different sites that simulate assisted migration along a climatic gradient. Furthermore, few tests dedicated to assisted migration have been established, such as the Assisted Migration Adaptation Trial (O'Neill et al., 2013), and the test developed by the ministère des Forêts, de la Faune et des Parcs du Québec (MFFP) (Lamhamedi et al., 2017). These assessments will permit identification, at a young age, of seed sources presently used in reforestation programs that possess the broadest genetic adaptation and the largest phenotypic plasticity, which should consequently perform better under future environmental conditions of planting sites. In tree plantations, the juvenile phase of establishment is considered to be the stage where the seedlings are the most sensitive to different environmental stresses (Lamhamedi and Bernier, 1994; Grossnickle, 2000). Our hypothesis is that under anticipated climate change, morpho-physiological traits will only be optimized in plantations by matching sites to seed sources from regions where the climatic conditions are currently similar to those predicted in future scenarios for the reforestation sites, since local genetic adaptation is significant in white spruce in eastern Canada (Andalo et al., 2005; Beaulieu and Rainville, 2005). The assessment of morpho-physiological traits in the context of assisted migration tests should contribute to a better characterization of the different seed sources with respect to their lag in adapting to local conditions, capacity to acclimatize, tolerance to environmental stresses, efficiency in using environmental resources (water, mineral elements etc.), and ability to sequester carbon. Over the long term, a better knowledge about the various levels of genetic adaptation and plasticity under various environmental conditions will be obtained.

This article is the fourth of a series of papers addressing the anticipated impacts of climate change on the adaptation of the most frequently used white spruce seed sources in Québec's reforestation program. Recent work has focused on morpho-physiological characterization under controlled conditions (greenhouse), in a nursery and during the first 2 years of establishment on reforestation sites (Benomar et al., 2015, 2016; Villeneuve et al., 2016). This characterization has revealed differences between seed sources (seed orchards) and established links between seedling performance and the climatic conditions of the original location of the parental trees that were sampled to provide grafts for orchard establishment (Villeneuve et al., 2016). The present study continues this investigation by examining these traits in experimental plantations established on a number of forest sites along a climate gradient. The study was conducted during the first few years after outplanting, which are generally the most critical for seedling survival.

To our knowledge, the present study is the first to address the contribution of genetic adaptation and phenotypic plasticity in response to a climatic gradient for traits such as photosynthesis before bud break, the allocation of carbon and biomass between different parts of the tree, and the evolution of the nutrient status of the seed sources as a function of site quality using vector analysis (Haase and Rose, 1995). The objectives were to: (1) model the evolution of juvenile growth of the different white spruce seed sources on three sites along a climatic gradient for the first 4 years after outplanting; (2) quantify the allocation of carbon and biomass of the different white spruce seed sources and the evolution of their nutrient status with respect to planting sites; and (3) evaluate the response of photosynthesis before bud break of the different white spruce seed sources on the three planting sites.

## Materials and methods

### Vegetative material

**Figure 1:**
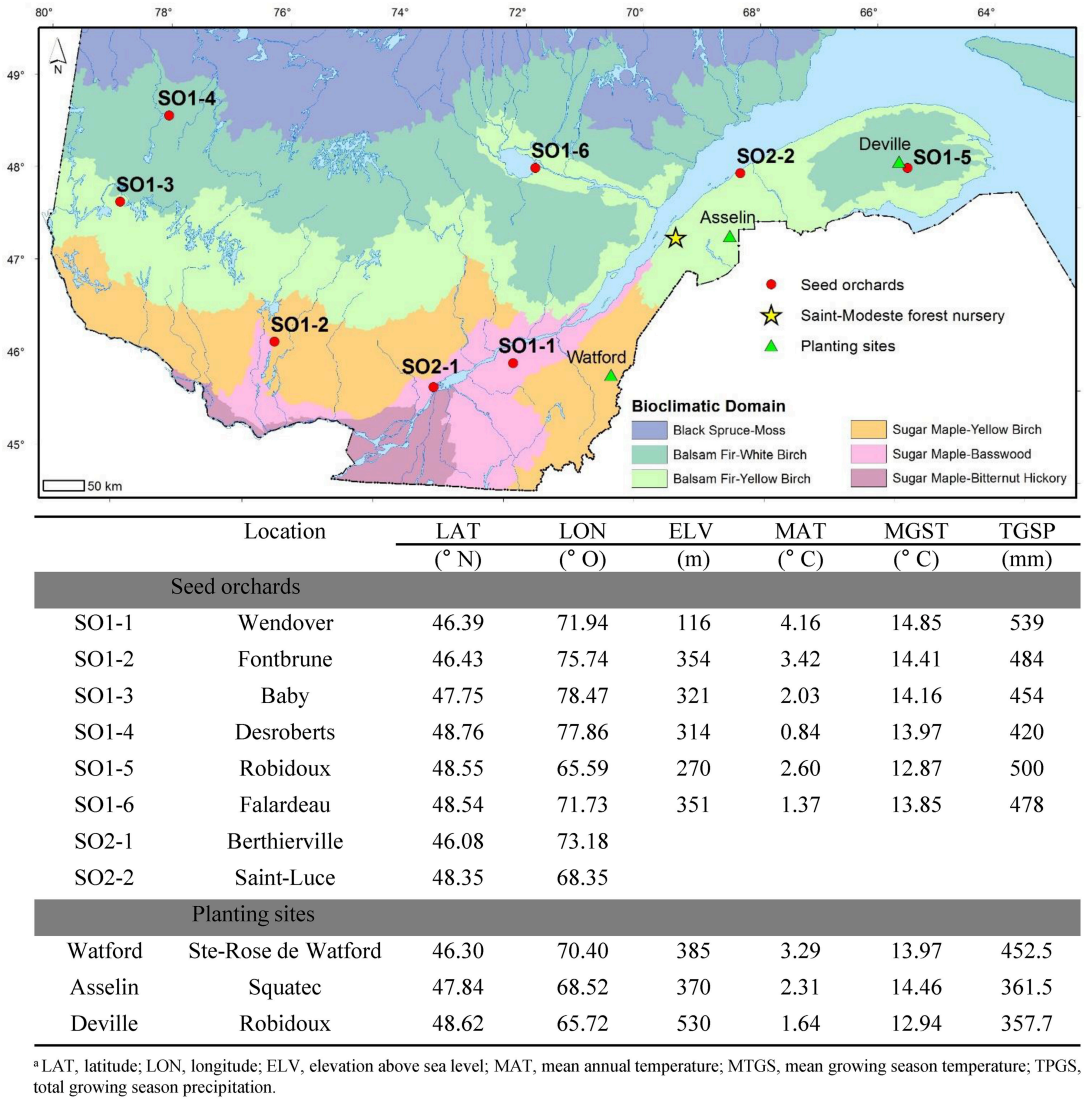
Map of Quebec showing the location, coordinates and climatic data for the eight white spruce seed orchards (SO) that were used as seed sources in this study as well as the three planting sites (Watford, Asselin, and Deville). For SO1-1 to SO1-6, latitude, longitude, and elevation of the seed orchards are those of the centroid of selected trees that make up the orchards. The climatic data corresponds to those of the same centroids for the period 1981–2010. For seed orchards SO2-1 and SO2-2, only latitudinal and longitudinal data of orchard locations are presented because the orchards were composed of trees representing multiple widespread provenances from Québec and Ontario in Canada.

The seed sources used in this study are representative of the most commonly used seed orchards in Québec's reforestation program. Of the eight sources, six represent first-generation clonal seed orchards (SO1-1 to SO1-6) and two represent second-generation clonal seed orchards (SO2-1 and SO2-2) (Figure 1). The first-generation seed orchards consist of plus-trees selected from local natural populations that were reproduced by grafting. They were intended to produce high quality seed to meet the needs of the surrounding regions (Beaulieu et al., 2009). The two second-generation orchards have origins covering a much larger geographical range and were designed to respond to the reforestation needs in the balsam fir and sugar maple forest domains, respectively, which constitute distinct zones of genetic adaptation for white spruce (Li et al., 1997a). These orchards are composed of trees selected for superior height, stem form and branching in progeny tests conducted at about 15 years of age and represent the 25 best-performing open-pollinated families in each of these two forest domains. Most of the families are common to both seed orchards and were originally from natural white spruce populations in Québec and Ontario. These seed orchards therefore possess a genetic composition representative of a larger geographic territory than the first-generation seed orchards.

The seeds used for seedling production in this study were extracted from cones harvested in two consecutive seed years (2008 and 2009), to obtain an optimal representation of the genetic pool of each seed orchard. The seeds were sorted by size and those with a diameter between 1.5 and 2.5 mm were stratified by le Centre de semences de Berthier (Berthier Seed Center; MFFP) following operational standards. Seed size has been shown to affect growth and mineral nutrition of white spruce seedlings (Lamhamedi et al., 2006b). Generally, seeds with diameters <1.5 mm have a slow rate of germination and the resulting seedlings do not meet the 28 morpholphysiological criteria (Veilleux et al., 2012) contractually required before delivery to reforestation sites. Following stratification, the seeds were germinated under a polyethylene production tunnel at the St-Modeste forest nursery (47.50° N, 69.23° W) (Figure 1), where the seedlings were grown under standard nursery cultural treatments during their first growing season (1+0) (2011) (Lamhamedi et al., 2001). The seedlings were moved outside for their second growing season (2+0) (2012). Irrigation was optimized in accordance with growth stage during the first growing season (Lamhamedi et al., 2001), and maintained between 40 and 45% (v/v) during the second growing season (Lamhamedi et al., 2006a). Fertilisation was adjusted bi-weekly using the PLANTEC software (Girard et al., 2001) during both growing seasons. The seedlings were planted on three reforestation sites (Watford, Asselin and Deville) in the spring of 2013 (Figure 1).

### Planting sites

The experimental design is composed of nine planting sites, distributed across the province of Québec. Three sites (Watford, Asselin and Deville), established in the spring of 2013 within three bioclimatic domains representative of a climatic gradient along the Appalachian range, were used for this study (Figure 1). Watford township, in the Beauce region, is located in the sugar maple-yellow birch domain and characterized by the most temperate climate (Saucier et al., 2010). Asselin Township is located in the Témiscouata region, within the balsam fir-yellow birch domain and has an intermediate climate. These two first sites are characterized by loamy soils. The third site is located in Deville Township on the Gaspé Peninsula, in the balsam fir-white birch domain and is characterized by sandy-loam soils. It is also the most northerly site of the three sites and has the harshest climate (Figure 1).

A total of 4,608 seedlings were planted on each site (density of 2,000 seedlings/ha with spacing of 2.25 × 2.25 m) and distributed in four randomized complete blocks. Each block consisted of eight plots, each occupied by one of the eight seed sources being evaluated. Each plot contained 144 seedlings (12 × 12). The seedlings in the two outside rows of each plot were excluded from the measurements in an effort to reduce the border effects.

### Climatic data

Climatic data on each of the three sites was registered continuously during the four growing seasons using weather stations. The stations were installed on May 27, 2013 (Watford), June 3, 2013 (Asselin) and June 11, 2013 (Deville). They were equipped with rain gauges, as well as probes to measure humidity (HMP50, Vaisala, Helsinki, Finland), air temperature (HMP45C, Campbell Scientific, Logan, UT, USA), and incoming light (PAR) (Li 190 Campbell Scientific, Logan, UT, USA). Soil temperature was also measured at three depths (10, 20, and 30 cm) using 107B probes (Campbell Scientific, Logan, UT, USA).

The gradient of average annual temperature over the three sites (1.7°C) and the climatic data from the seed orchards of origin were derived from simulations generated by BioSim software (Régnière et al., 2014) over a period of 30 years (1981–2010) using climate data collected in neighboring weather stations.

### Characterization of the soil on the planting sites

Ten random soil samples were collected on each of the three planting sites (total of 30 samples) to obtain a fair representation of the spatial variability of soil characteristics. After removal of the superficial layer of organic matter, samples were taken from the top 20 centimeters of soil using an auger. These samples were used to determine soil particle size, texture and fertility on each of the study sites. After sieving (2 mm) and air drying, a portion of each sample was ground using a Pulverisette-7 (Fristch, Idar-Oberstein, Germany) to determine concentrations of mineral nutrients (N, P, K, Ca, Mg) and micro-nutrients. Nitrogen analysis was conducted using the high combustion method (LECO analyser, Leco Corporation, St-Joseph, Michigan) (Yeomans and Bremner, 1991). An exaction with a Mehlich-3 solution permitted separation of the other mineral nutrients and micro-nutrients (Ziadi and Tran, 2006). The concentration of each of the mineral nutrients was then determined using plasma emission spectrometry. Cationic exchange capacity (CEC) and pH of the soil were also evaluated (Baize, 2000).

### Initial characterization of white spruce seedlings

Before outplanting, seedlings were characterized in May 2013 (Villeneuve et al., 2016). Data was collected on 15 seedlings/seed orchard for height and diameter growth. Moreover, three composite samples per seed orchard (five seedlings per composite sample) were used to determine the nutrient content (concentration ^*^ dry mass) of the shoot and root tissues.

### Growth, root morphology and allocation of biomass

Seedling height and diameter growth from the different seed orchards were evaluated each year during the 4-year study (2013–2016). The mortality and presence of dead apical buds was also noted. All measurements were made on the 64 seedlings at the center of each plot. A total of 6,144 seedlings (64 seedlings ^*^ eight seed orchards ^*^ four blocks ^*^ three sites) were evaluated in 2013, 2014, and 2015. In 2016, only 63 of the 64 seedlings were measured, since one seedling from each plot (one seedling ^*^ eight orchards ^*^ four blocks ^*^ three sites = 96 seedlings in total) was removed at the beginning of the growing season for laboratory analysis of growth and mineral nutrition.

Ninety-six seedlings were also destructively sampled before bud break in May 2016 for assessment of root morphology and allocation of biomass. The position of the first seedling sampled was randomly selected in the first plot. Other seedlings were systematically selected from the same relative position in the other plots and blocks for each of the three sites. Care was taken to conserve the majority of the root system during harvesting. The seedlings were taken to the MFFP laboratory where they were cleaned with compressed air and washed to remove all traces of soil and debris. The root systems of the 96 white spruce seedlings were photographed in their entirety before being oven dried at 63°C for 3 days. The root systems were then trimmed of fine roots and shortened to expose the main secondary and adventitious roots. The 32 seedlings harvested from each of the three sites were used to conduct a qualitative analysis following the criteria established by Gingras et al. (2002). In addition, an assessment of the presence of adventitious roots (1 or 0) and the differentiation between normal (0) and deformed (1: J-shaped, 2: L-shaped, 3: twisted, 4: swollen) root systems was conducted. The horizontal distribution of the roots was also evaluated by differentiating between a system that was equally distributed on each side of a central axis (0), tended toward one side (1) or completely oriented to one side (2). Finally, the degree of persistence of the plug root system form was noted as poor (0), moderate (1), or strong (2).

Total height, diameter and first-order lateral branches as well as the numbers of adventitious roots, were determined for each of the seedlings. After separation, the constituent parts (adventitious roots, roots, shoots without needles, needles) of each seedling were oven dried at 63°C for 3 days before determining their dry masses with a precision balance (0.1 g).

### Mineral nutrition and carbon allocation

Mineral nutrient analyses were conducted on the same seedlings used for determination of dry masses (roots, shoots without needles, and needles). Pre-grinding (grinder model: Willy-Mill #3. Arthur H. Thomas Co., Philadelphia) was used to reduce the size of the large roots and the main stems. The constituent tissues (roots, shoots without needles, needles) were then subjected to a finer grinding before analyses. The carbon concentration of the woody tissues, roots and needles was determined by the same process used for soil nitrogen, which is high combustion. However, once the carbon was oxidized, its presence was determined using infrared detection rather than thermal conductivity (Yeomans and Bremner, 1991).

The nutritional status of the different seed sources on the three sites was evaluated in 2016 using vector analysis (Munson and Bernier, 1993; Haase and Rose, 1995). The values for nutrient status and seedling growth for each seed orchard, as determined during the pre-planting characterization in 2013, were used as reference points.

### Gas exchange

Gas exchange measurements were conducted before bud break (early May 2016) for each seed source on each site. Average air and soil (20 cm depth) temperatures, 5 days prior to gas exchange measurements were 8.2 and 5.5°C respectively, for Watford, 8.9 and 8.1°C for Asselin, and 7.1 and 6.5°C for Deville. Gas exchange was measured on 1-year-old needles using a portable infrared gas analyser LI-6400 (Li-Cor Inc. Lincoln, NE). Buds were removed before each measurement (Benomar et al., 2015), so that only the net photosynthesis (An) and stomatal conductance (g_s_) of the needles was captured. The measurements were conducted under optimal environmental conditions inside a chamber (temperature of 20°C, relative humidity of 60 ± 10%, constant vapor pressure deficit (VPD) of 1.0 ± 0.2 kPa, and saturated light intensity of 1,000 μmoles/m^2^/s). To determine the level of saturated photosynthetically active radiation (PAR), light response curves were produced for six seedlings from seed orchards SO1-1 and SO1-5 (3 seedlings per orchard). The obtained values were similar to those previously reported (Lamhamedi et al., 2003; Benomar et al., 2015). Gas exchange measurements were conducted on a total of 96 randomly selected seedlings (one seedling ^*^ 8 seed orchards ^*^ 4 blocks ^*^ 3 sites). The branches used for the measurements were excised and conserved at −20°C for future determination of the projected foliar surface area and subsequent adjustments to the photosynthetic rate.

### Specific leaf area, nutrient use efficiency (NUE) and water use efficiency (WUE)

The projected surface area of the needles, as well as their length (mm) and width (mm), were measured using the WinSeedle (Version 2007 Pro. Regent Instruments, Québec, Canada) image analysis software. The needles were then dried at 63°C for 3 days and subsequently weighed. The specific leaf area (SLA) corresponds to the relationship between projected surface area (cm^2^) and needle dry mass (g).

Other branches previously cut from the same whorl used for photosynthesis measurements were harvested, ground and subjected to high temperature combustion (Leco Corporation. St-Joseph. Michigan) for determination of foliar nitrogen and phosphorus concentrations and estimation of photosynthetic nitrogen-use (PNUE) and phosphorus-use efficiencies (PPUE). These latter two measurements correspond to the relationship between photosynthetic capacity (A_max_) and leaf nutrient concentration/unit area (N_area_, Nitrogen; P_area_, Phosphorus). Intrinsic water-use efficiency (WUE) is defined as the ratio of A_max_ to g_s_.

### Statisitical analyses

Soil characteristics of the three sites were analyzed using a linear model (model 1) with the MIXED procedure of SAS (version 9.4, SAS Institute, Cary, NC, USA) where site was considered a fixed effect factor and the block within the sites as a random effect factor.

Growth traits and mineral nutrition 4 years after outplanting, as well as photosynthetic traits before bud break in the fourth year were also analyzed with the MIXED procedure using the average values for each experimental unit (plot corresponding to a single combination of orchard/block/site), where site, seed orchard, and their interaction were treated as fixed effect factors and the block as a random effect factor. When the seed orchard effects were significant at the 5% significance level, contrast analyses were conducted to test whether there was a significant difference between the first- and second-generation seed orchards.

(1) $$y_{ijk}=\mu +\beta_{i}+\gamma_{j}+\beta \gamma_{ij}+b_{k(i)}+e_{ijk}$$

Where *Y*_*ijk*_ is the dependant variable, μ is the grand mean, β_*i*_ is the fixed effect of site *i*, γ_*j*_ is the fixed effect of seed orchard *j*, βγ_*ij*_ is the fixed effect due to the interaction between the site i with the seed orchard *j*, *b*_*k*(*i*)_ is the random effect of the block *k* within the site *i* and *e*_*ijk*_ is the residual error.

When required, Tukey's multiple comparison tests were used to assess differences while taking into account multiple testing. When the interaction was significant, seed orchard means were compared within each of the sites. The assumptions of normality and homogeneity of variance were verified by graphical analyses of the residuals. When they were not satisfied, the data were transformed. However, if only the hypothesis of normality was not met, a non-parametric analysis (rank test) was conducted, confirming, in all cases, the results of the parametric analysis.

For height growth, a repeated measurements model was applied to the mean values of each experimental unit (plot) at the end of each growing season (2013–2016). In this model, years, seed orchards, planting sites and their interactions were considered as fixed effect factors and the blocks nested within the sites and their interaction with seed orchards were considered as random effect factors. A variance-covariance structure was used to take into account the autocorrelation existing between measurements made on the same experimental unit. Because the variance increased with time, structures considering a different variance for each assessment time were tested. The Toeplitz heterogeneous covariance structure (TOEPH) (Littell et al., 2006) was finally retained. Weighs corresponding to inverse variances of combinations of site-time was also used, since the variance increased differently with time for each site. This procedure also permitted to validate the assumptions of normality and homogeneity of variance without having to transform data. Contrast analyses made also possible to verify height differences over time between seedlings originating from first- and second-generation seed orchards.

A non-linear logistic growth model was selected to model the juvenile growth, because it was the most appropriate for representing the observed values over the time period of the study (4 years). Growth curves were modeled for each of the sites using the SAS NLMIXED procedure, as preliminary analyses showed that there was no significant difference between the curves of the different seed orchards at each site. The following model was employed:

(2) $${\bar{h}}_{ijkl.}=\frac{a_{i}}{1+e^{-c_{i}(temps_{l}-b_{i})}}+\beta_{j(i)k}+\varepsilon_{ijkl.}$$

where ${\bar{h}}_{ijkl}$ is the average height of the seedlings from seed orchard *k* situated in block *j* on site *i* at time *l*; *a*_*i*_, the final height (asymptote of the curve) for site *i*; *b*_*i*_, time at which half of the final height was attained (inflection point) for the site *i*; *c*_*i*_, the rate of growth for the site *i*; *e*, the base of the natural logarithm function; β_*j*(*i*)*k*_, the random effect of the experimental unit corresponding to the seedlings from the seed orchard *k* situated in block *j* of site *i* and ε_*ijkl*._, the residual error.

Pearson correlation coefficients were estimated to test the relationship between the means of the initial (end of the 2012 growing season in a forest nursery) and 2016 growth data, as well as the association between photosynthetic traits before bud break in the fourth year, using the SAS CORR procedure.

Finally, the SAS GLIMMIX procedure was used to verify the differences among the average values for sites, seed orchards and their interaction for survival traits and the presence of adventitious roots, the persistence of the plug root system form, the presence of abnormal root system orientation and deformed roots, considering the binary distribution and the logit link function.

## Results

### Soil characterization

The majority of soil fertility and chemical properties exhibited significant differences among sites, with the exception of carbon (*P* = 0.15), phosphorus (*P* = 0.58), potassium (*P* = 0.12), zinc (*P* = 0.11) and sulfur (*P* = 0.42; Table 1). Soil pH (*P* = 0.01), nitrogen (*P* < 0.0001), calcium (*P* = 0.01), magnesium (*P* = 0.05), manganese (*P* = 0.05), and sodium (*P* = 0.0002) were all significantly higher at Watford than at Asselin and/or Deville (Table 1). Aluminium (*P* = 0.001) and C.E.C_total_ (*P* = 0.02) were significantly lower at Watford than at Asselin and Deville, whereas iron (*P* = 0.003) at Watford was only lower than at Asselin. The Watford and Asselin sites had similar average soil temperatures at 20 cm depth throughout the four growing seasons. However, the soil at Deville was 2°C cooler.

**Table 1:** Comparison of soil characteristics of the three planting sites located along a climatic gradient.

**Traits**	**Sites**		
	**Watford (south)**	**Asselin (intermediate)**	**Deville (north)**
pH_CaCl2_	4.50 ± 0.31a^*^	3.89 ± 0.28b	3.89 ± 0.16b
C.E.C_total_ (méq/100 g)	25.97 ± 2.15b	33.24 ± 2.53a	34.61 ± 7.38a
Carbon (g/kg)	41.60 ± 5.42a	30.88 ± 7.53a	33.63 ± 9.61a
Nitrogen (g/kg)	3.14 ± 0.37a	2.29 ± 0.25b	1.93 ± 0.38b
Phosphorus (mg/kg)	12.10 ± 5.26a	12.13 ± 8.98a	9.125 ± 3.36a
Potassium (mg/kg)	76.90 ± 27.93a	55.25 ± 17.18a	53.63 ± 10.17a
Calcium (mg/kg)	608.90 ± 455.15a	169.71 ± 170.12ab	79.90 ± 42.95b
Magnesium (mg/kg)	34.30 ± 12.40a	23.57 ± 12.32ab	18.75 ± 7.27b
Manganese (mg/kg)	19.10 ± 9.11a	7.29 ± 4.07b	10.25 ± 4.40ab
Aluminium (mg/kg)	1586.00 ± 198.06b	1995.00 ± 383.88a	2305.00 ± 245.59a
Iron (mg/kg)	233.20 ± 66.10b	452.25 ± 67.71a	331.25 ± 93.63ab
Zinc (mg/kg)	1.76 ± 0.84a	2.50 ± 1.07a	1.58 ± 0.80a
Sodium (mg/kg)	8.00 ± 4.31a	5.86 ± 6.13b	4.86 ± 3.64b
Sulphur (mg/kg)	17.10 ± 4.31a	14.13 ± 6.13a	14.87 ± 3.64a

* *For a given row, the means (n = 10), followed by their standard errors, having the same letter are not significantly different according to a Tukey test at α = 0.05*.

### Growth characteristics of progeny from white spruce seed orchards 4 years after planting

At the end of the fourth growing season, overall survival was 95%. When data for all orchards and sites were combined, the average seedling height was 112.4 ± 23.6 cm and the average root collar diameter was 24.3 ± 5.0 mm. No site ^*^ seed orchard ^*^ year interaction (*P* = 0.66) was found for height over time, although site ^*^ year and seed orchard ^*^ year interactions both showed a significant effect (*P* < 0.0001). No site ^*^ seed orchard interaction was found for any of the other growth traits analyzed (Table S1).

**Figure 2:**
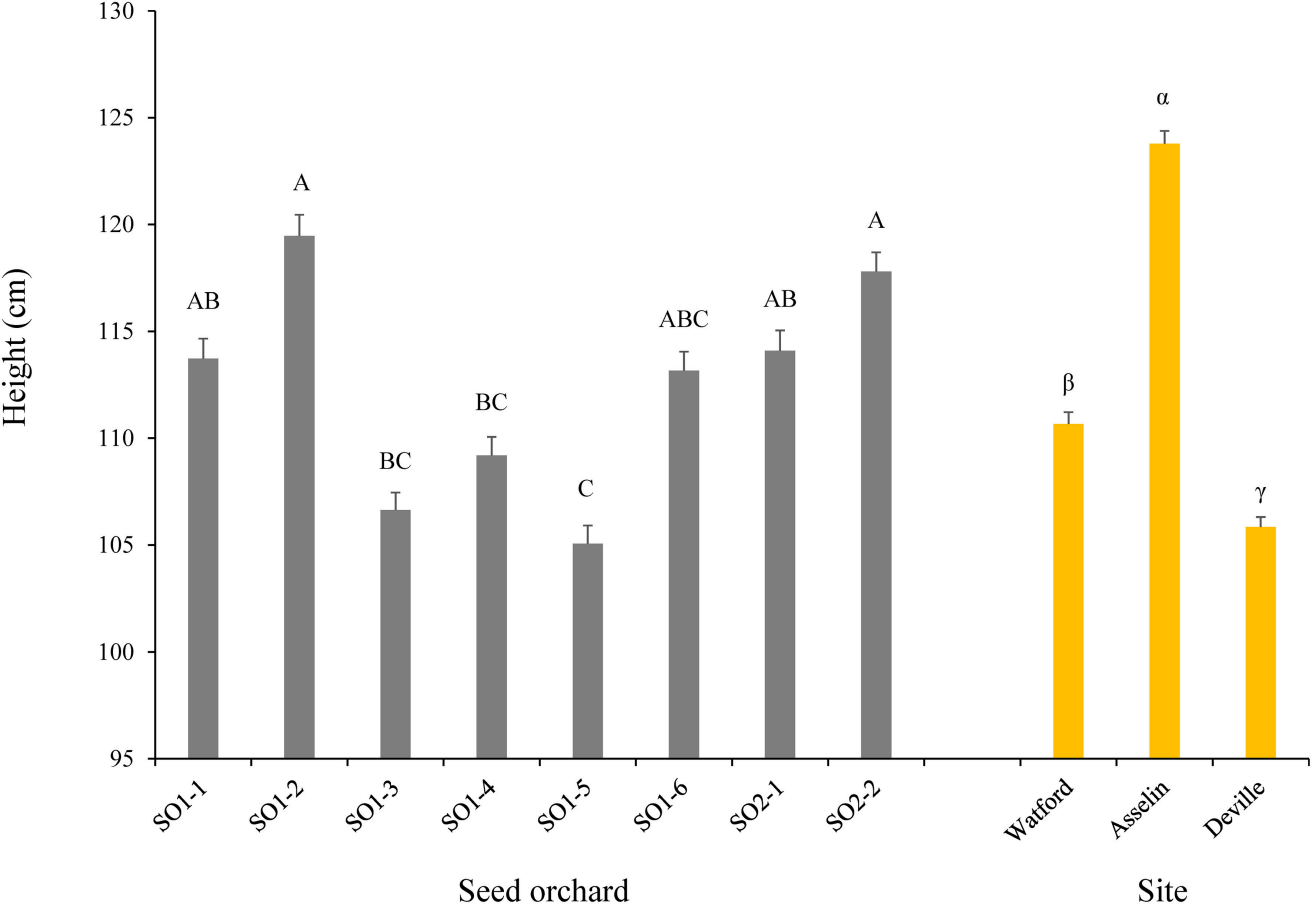
Plant height as a function of seed orchard and forest planting site at the end of the fourth growing season (*n* = 768 seedlings per seed orchard and *n* = 2,048 seedlings per site for a total of 6,144 seedlings). The means (± standard error) with the same letter within seed orchard or site are not significantly different according to a Tukey test at α = 0.05.

**Figure 3:**
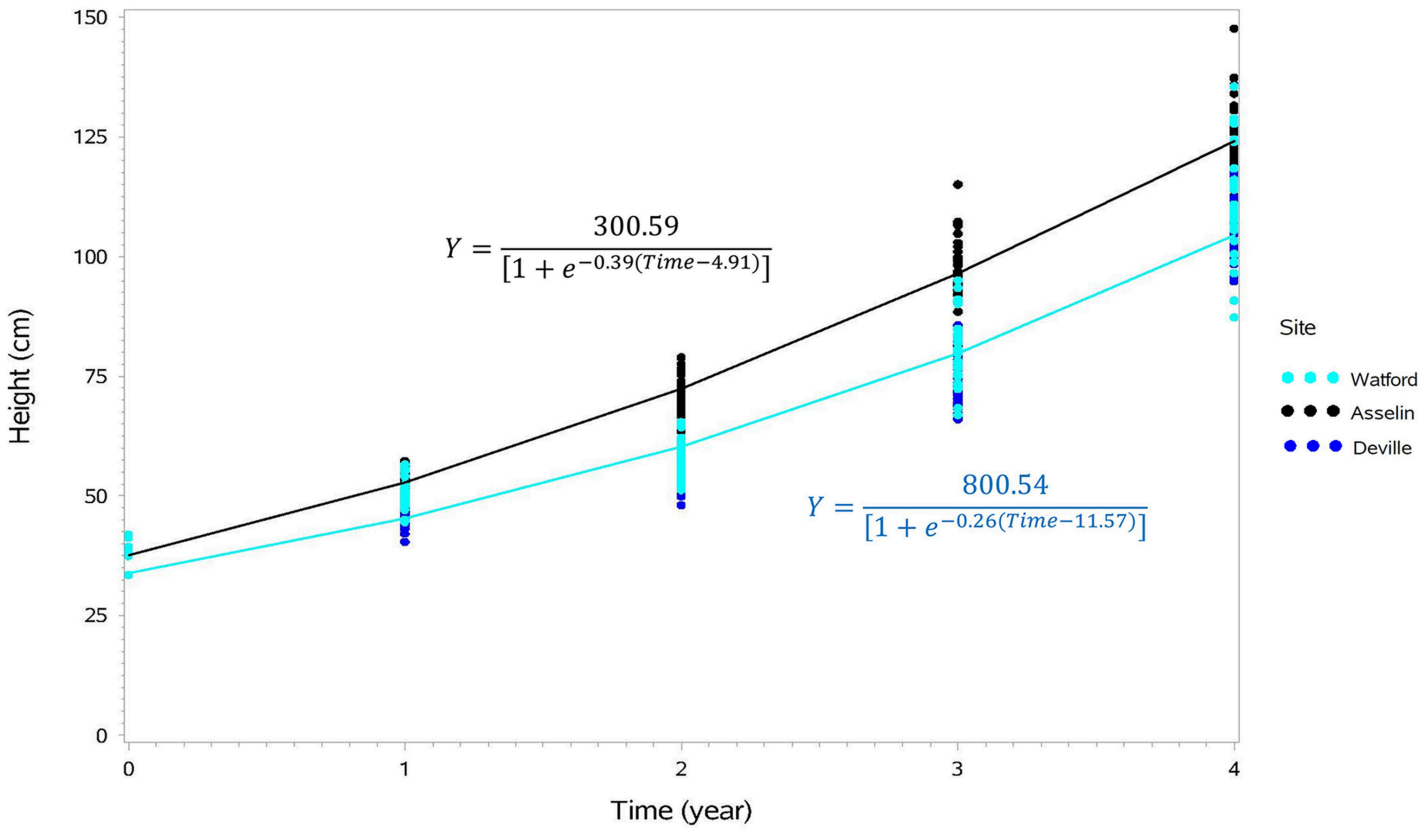
Cumulative height growth curves representing the growth of seedlings from eight seed orchards on the Watford (south), Asselin (intermediate), and Deville (north) forest sites during four growing seasons after outplanting, and their associated equations (Asselin site in black and the Watford and Deville sites in blue, not significantly different). Each point for time 0 corresponds to the average for each seed orchard (*n* = 15) just before planting and each other point represents the average of an experimental unit (orchard/block/site; *n* = 63).

**Figure 4:**
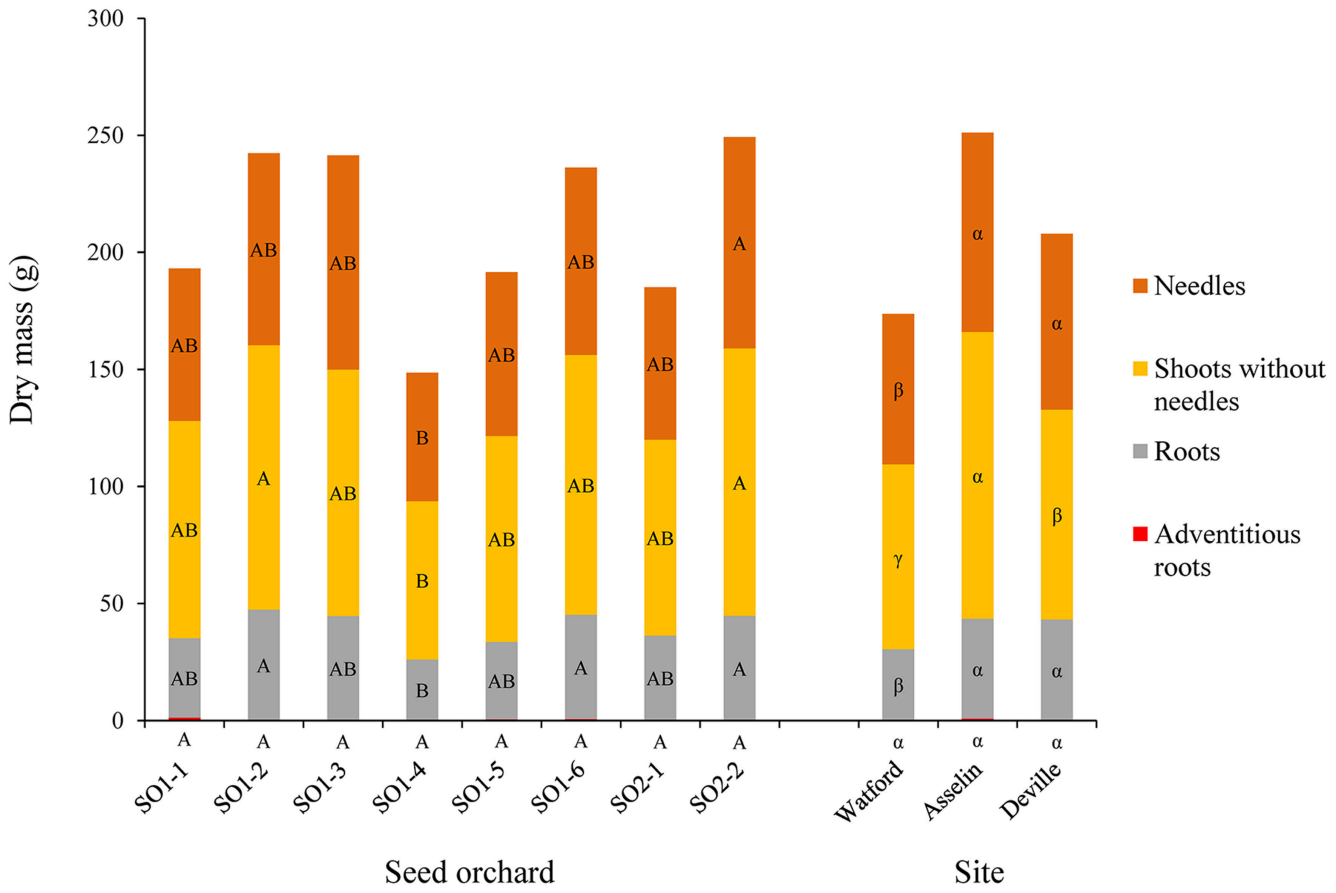
Mean distribution of the dry mass from the seedling parts (adventitious roots, roots, shoots without needles and needles) as a function of seed orchard and planting site (*n* = 96). For the same part of the seedling, the means with the same letter within seed orchard or site are not significantly different according to a Tukey test at α = 0.05.

Tree survival was significantly different among the three sites where Deville was the highest (99.6%), followed by Watford (95.9%) and Asselin (88.6%). The analysis of variance indicated a significant site effect for height (*P* < 0.0001) over time. The sites maintained the same ranking over the 4 years, where Asselin, which was intermediate in terms of climate and geography, showed the best height growth, followed by Watford (more southerly) and Deville (more northerly) (Figure 2). Comparison of the parameters *a* (asymptote), *b* (inflection point) and *c* (growth rate) of the logistical height growth models for each site showed that the models for the Watford and Deville sites did not differ significantly for the three parameters. Therefore, a new model was created to generate two height growth curves, one for Asselin and one for Watford and Deville combined (Figure 3), and showed a significant difference between the curves for the three parameters. With the exception of diameter (*P* = 0.574, results not shown) and dry mass of adventitious roots (*P* = 0.30), all other growth traits (dry mass of roots (*P* < 0.0001), shoots without needles (*P* < 0.0001), needles (*P* = 0.01), and R/S ratio (*P* < 0.0001, results not shown) and total seedling dry mass (*P* < 0.0001) were significantly different between sites (Figures 2, 4).

No significant difference was noted between first and second-generation seed orchards for any of the growth traits except height (*P* = 0.0004). Contrasts showed that, on average, seedlings from second-generation seed orchards were significantly taller than those from first-generation seed orchards. After 4 years in plantation, the seedlings from the second-generation seed orchards exhibited an average height of 115.9 ± 23.5 cm, while those from the first-generation seed orchards had an average height of 111.2 ± 23.8 cm. The orchard ranking remained essentially the same throughout the 4-year plantation study. Only the seedlings from seed orchards SO1-6 and SO2-1, which were significantly shorter than those of seed orchards SO1-2 and SO2-2 1 year after outplanting, increased slightly in rank, and did not differ significantly from those from the same seed orchard at the end of the study. After four growing seasons, seedlings from seed orchards SO1-2 (119.5 ± 25.3 cm) and SO2-2 (117.8 ± 23.1 cm) were significantly taller than those from SO1-3 (106.6 ± 21.1 cm), SO1-4 (109.2 ± 22.6 cm), and SO1-5 (105.1 ± 21.8 cm). The effect of seedling origin (seed orchard) was also significant for all other growth traits studied, except for dry mass of adventitious roots (*P* = 0.21) and R/S ratio (*P* = 0.64). The seedlings from seed orchard SO1-2 (26.1 ± 4.6 mm) had average diameters significantly larger than those from seed orchards SO1-1 (23.9 ± 5.0 mm), SO1-3 (23.6 ± 4.2 mm), SO1-4 (23.1 ± 4.9 mm), and SO1-5 (23.78 ± 4.3 mm).

The correlation coefficients between average values before outplanting and at the end of the current study for the various seed orchards were significant for both height (*P* = 0.02) and diameter (*P* < 0.001), with positive Pearson correlation coefficients of 0.47 and 0.63, respectively. No significant correlations were observed between initial and final dry mass values.

With regard to root morphology, the proportion of seedlings with adventitious roots was 31.3%. No significant site ^*^ seed orchard interaction or differences between sites or seed orchard were observed for the presence of adventitious roots, as well as for orientation, deformation and persistence of the plug system form (Figure S1). In addition to the dry mass, the root systems of the seedlings grown at Asselin and Deville also seemed to have more fine roots than those grown at Watford (Figure S2).

### Mineral nutrition and evolution in nutrient status

**Figure 5:**
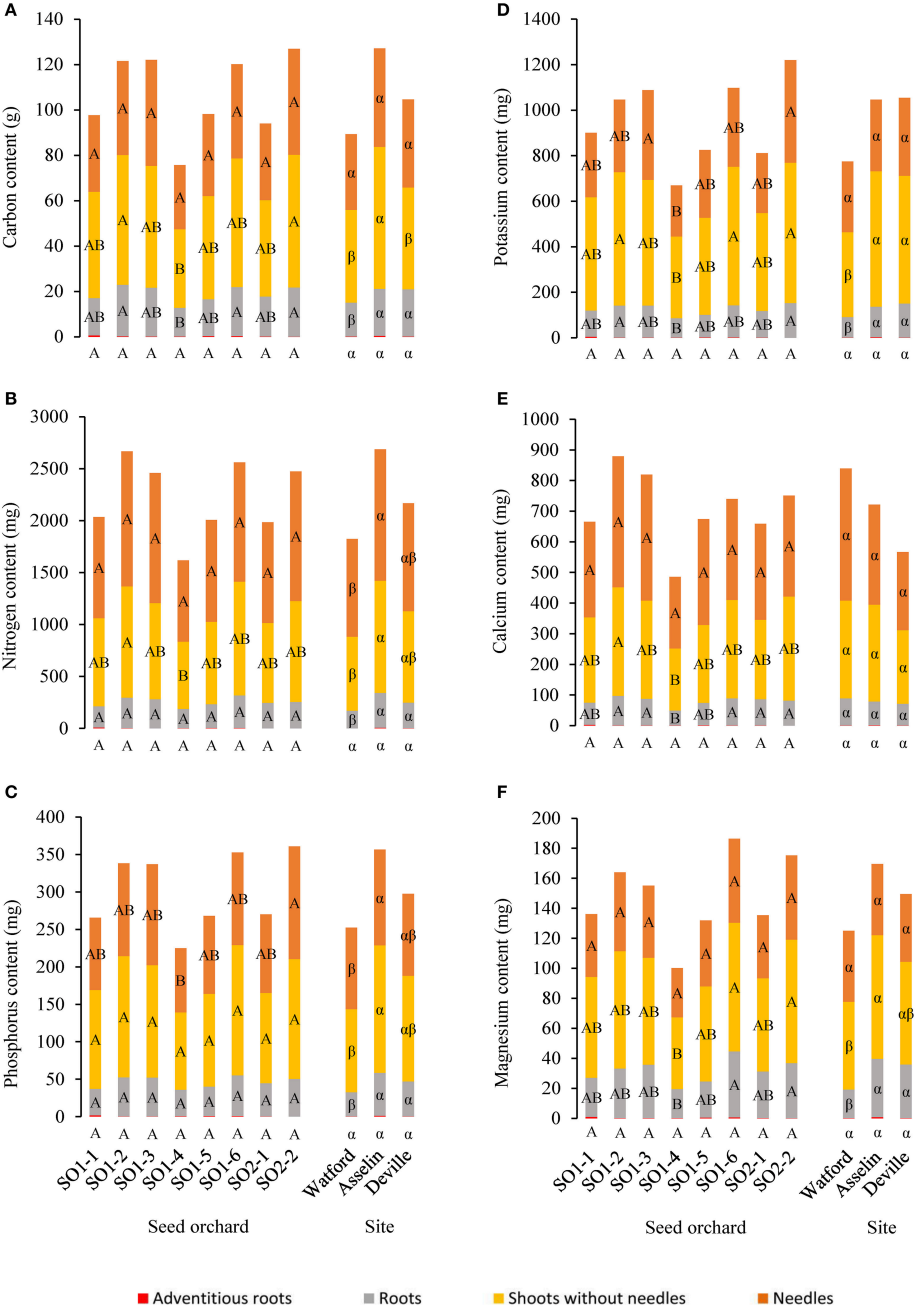
Average distribution of nutrient content between seedling parts (adventitious roots, roots, shoots without needles and needles) as a function of seed orchard and planting site (*n* = 96) for **(A)** carbon, **(B)** nitrogen, **(C)** phosphorus, **(D)** potassium, **(E)** calcium, and **(F)** magnesium contents. For the same seedling part, means with the same letter within seed orchard or site are not significantly different according to a Tukey test at α = 0.05. The content was based on dry mass.

No significant interaction was observed between planting site and seed orchard for carbon and all other mineral contents and concentrations measured in the different parts of seedlings. All mineral contents in seedlings showed a significant effect of site, with the exception of adventitious roots for all nutrients (*P* = 0.17), Ca content in roots (*P* = 0.31), needles (*P* = 0.06) and shoots without needles (*P* = 0.16) and Mg (*P* = 0.52), K (*P* = 0.31), and C (*P* = 0.08) in the needles (Figure 5). Apart from calcium, the general tendency indicates a higher nutrient content in constituent parts of seedlings grown at Asselin compared with those from Watford. In terms of mineral concentrations (content per dry weight) in roots of the different seedlings, each element differed significantly among sites. Compared to other sites, seedlings from Asselin had higher N and P concentrations, those from Watford showed higher Ca and lower Mg concentrations and Deville seedlings were higher in K. For the needles, no significant difference among sites was noted for N and P, which had average concentrations of 14.6 ± 2.2 and 1.5 ± 0.2 mg/g, respectively. Foliar K concentration from Asselin (3.7 ± 0.4 mg/g) was significantly lower (*P* = 0.0004) than those from Deville (4.5 ± 0.8 mg/g) and Watford (4.8 ± 0.9 mg/g).

A significant effect of seed orchard was observed for all mineral element contents in the roots except N (*P* = 0.10) and P (*P* = 0.13), for all of the mineral elements in the shoots without needles except P (*P* = 0.07), and for foliar P (*P* = 0.02) and K (*P* = 0.01) (Figure 5). The general tendency indicated that seed orchards SO1-2, SO1-3, SO1-6 and SO2-2 had higher nutrient contents than those from SO1-4. In terms of mineral concentrations in roots of the different seedlings, no nutrient element showed a significant difference among seed orchards. For the needles, no significant difference among seed orchards was noted for N and P, while K concentration of seedlings originating from seed orchard SO2-2 (5.0 ± 1.1 mg/g) was significantly higher (*P* = 0.02) than those from seedlings originating from seed orchards SO1-4 (4.1 ± 0.8 mg/g) and SO1-2 (4.0 ± 0.7 mg/g).

**Figure 6:**
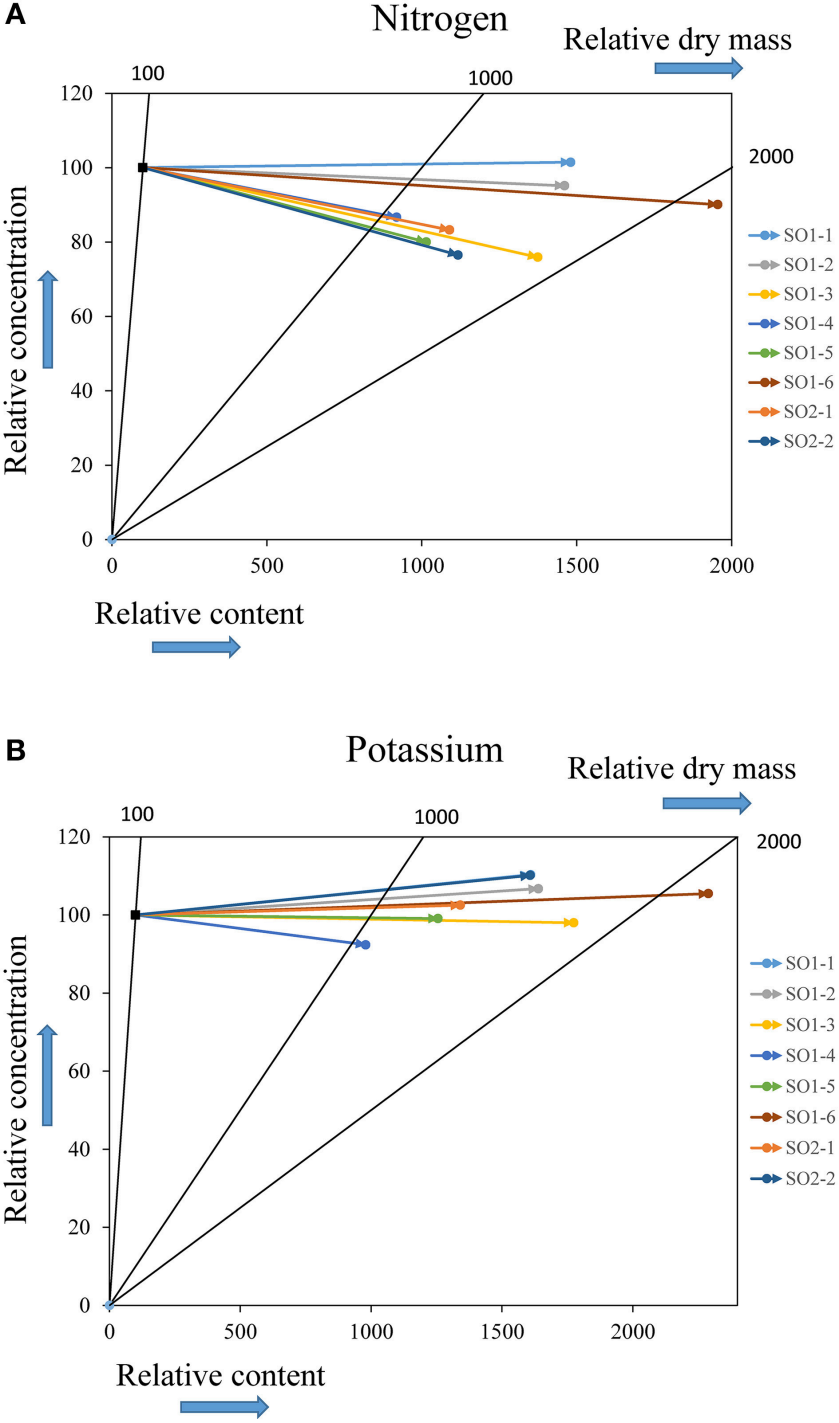
Vector analysis of the evolution of nutritional status for nitrogen **(A)** and potassium **(B)** in above-ground parts of seedlings from each seed orchard growing at the northerly site of Deville. The reference point (100) represents the initial characterization just before outplanting, whereas the final coordinates of each vector represent the nutritional status of seedlings from each seed orchard after four growing seasons on the planting site with respect to the reference point.

Vector analysis showed that since the time of planting, the evolution of the nutritional status within each site generally exhibited a dilution or sufficiency of mineral elements (N, P, K, Ca, Mg) in above-ground seedling parts. These dilution effects were greatest in Asselin seedlings and only seedlings from Deville showed effects of sufficiency (SO1-1 for N) or a slight deficiency (SO1-1, SO1-2, SO1-6, and SO2-2 for K) (Figure 6).

### Photosynthetic traits before bud break

None of the photosynthetic traits measured before bud break during the fourth growing season showed any significant site ^*^ seed orchard interaction. The effect of site was significant for the majority of traits (Table 2), except for A_max_ (*P* = 0.09), PPUE (*P* = 0.17), WUE (*P* = 0.31), and needle width (*P* = 0.06). Only specific leaf area (SLA) differed between seed orchard (*P* = 0.03), while seedlings from seed orchard SO1-5 (34.3 ± 5.2 cm^2^ g^−1^) had a significantly superior SLA to that of SO1-4 (29.6 ± 3.9 cm^2^ g^−1^).

**Table 2:** Difference in the level of photosynthetic traits of seedlings from the eight seed orchards as a function of the three planting sites located along a climatic gradient.

**Traits^a^**	**Sites**		
	**Watford (south)**	**Asselin (intermediate)**	**Deville (north)**
A_max_ (μmol CO^2^ m^−2^ s^−1^)	8.3 ± 1.4a	9.5 ± 1.5a	9.7 ± 1.0a
g_s_ (mmol m^−2^ s^−1^)	0.09 ± 0.02b	0.11 ± 0.02ab	0.12 ± 0.02a
SLA (cm^2^ g^−1^)	30.3 ± 2.5b	29.5 ± 3.4b	35.2 ± 3.9a
PNUE (μmol CO2 g^−1^ N s^−1^)	2.0 ± 0.4ab	1.8 ± 0.3b	2.4 ± 0.5a
PPUE (μmol CO^2^ g^−1^ P s^−1^)	16.1 ± 3.9a	14.3 ± 2.8a	16.7 ± 3.2a
WUE (μmol CO^2^/mol H_2_O)	91.7 ± 20.6a	89.7 ± 8.1a	80.6 ± 12.9a
DM (g)	0.50 ± 0.10a	0.55 ± 0.08a	0.37 ± 0.09b
Length (mm)	12.9 ± 1.9b	15.2 ± 2.4a	13.3 ± 2.5ab
Width (mm)	0.90 ± 0.06a	0.91 ± 0.07a	0.80 ± 0.10a
N (g/kg)	12.6 ± 1.3b	15.5 ± 1.5a	14.5 ± 2.5a
P (g/kg)	1.6 ± 0.3b	2.1 ± 0.3a	2.0 ± 0.2a
K (g/kg)	6.1 ± 0.9a	5.3 ± 0.7b	6.4 ± 0.7a

a *Trait abbreviations: A_max_, photosynthetic capacity; g_s_, stomatal conductance; SLA, specific leaf (needle) area; PNUE, photosynthetic nitrogen use efficiency; PPUE, photosynthetic phosphorous use efficiency; WUE, water use efficiency; DM, needle dry mass; Length, average needle length; Width, average needle width; N, nitrogen concentration in needles; P, phosphorus concentration in needles; K, potassium concentration in needles. For the same line, the means followed by the same letter are not significantly different according to a Tukey test at α = 0.05*.

The correlation coefficients estimated between these various photosynthetic traits showed that several of these were significantly correlated as well as with the seedling height observed at the end of the fourth growing season (Table 3).

**Table 3:** Pearson correlation coefficients (r) ^a^ of average seed orchard photosynthetic traits measured after the fourth growing season on three planting sites located along a climatic gradient (*n* = 24).

	**h_2016_**	**A_max_**	**G_s_**	**SLA**	**PNUE**	**PPUE**	**WUE**	**DM**	**Length**	**Width**	**N**	**P**
A_max_	0.08											
g_s_	−0.12	**0.77**										
SLA	−**0.61**	0.14	0.39									
PNUE	−**0.62**	0.33	**0.59**	**0.76**								
PPUE	−**0.55**	0.05	0.23	**0.62**	**0.76**							
WUE	0.08	−0.30	−**0.81**	−0.39	−**0.48**	−0.18						
DM	**0.65**	−0.39	−**0.53**	−**0.78**	−**0.76**	−**0.52**	0.33					
Length	**0.54**	0.23	0.07	−0.20	−0.16	−0.14	0.04	**0.43**				
Width	**0.44**	−0.22	−0.22	−**0.73**	−**0.54**	−**0.51**	0.06	**0.58**	0.17			
N	0.37	**0.57**	0.27	0.03	−0.34	−**0.42**	−0.02	−0.03	0.28	−0.13		
P	0.04	**0.72**	**0.62**	0.31	0.16	−0.34	−0.35	−**0.44**	0.06	−0.25	**0.74**	
K	−**0.64**	−0.08	0.24	**0.58**	**0.58**	0.32	−0.30	−**0.56**	−**0.50**	−**0.45**	−0.33	0.14

a *Significant correlations are in bold (P < 0.05). h_2016_ corresponds to the seedling height at the end of the 2016 growing season; the other abbreviations are described in Table 2*.

## Discussion

With predicted climate change for boreal forest, trees will likely lack ability to adapt or migrate, and become maladapted to the new conditions. Assisted population migration may help local populations to adapt to climate change by relocating them to environments experiencing climatic conditions similar to those to which they were adapted. The evaluation of eight seed sources on three planting sites located along a climatic gradient made it possible to simulate the effect of different climatic transfers. Through this study of morpho-physiological traits of young seedlings, it was possible to gain knowledge on the contribution of genetics and plasticity, during the critical phase of stand establishment, on the variation of traits such as growth (height and diameter), biomass and nutrition allocation, nutrient status and photosynthesis before budbreak.

No interaction between sites and seed orchards was found for any of the studied traits, indicating that seed sources had similar phenotypic plasticity. Photosynthetic-related traits before budbreak were similar among seed orchards and did not allow explaining the observed variation in growth along the climatic gradient. Height growth varied clinally with better performance of southern seed sources in each site. Use of southern seed orchards and second generation seed orchards should help improve plantation productivity in northern environment under climate change.

### Growth and performance of different white spruce seed sources grown under contrasted environmental conditions

The average survival rate (95%) of the seedlings from the different seed orchards after four growing seasons on the three planting sites was very high, indicating a good seedling establishment capacity and good tolerance to environmental stresses, even for the most northerly site, Deville. The height growth of the seedlings showed a negative relationship with latitude of the planting sites, which is in accordance with past observations for white spruce (Li et al., 1997a; Lu et al., 2014). When first-generation seed orchards representing distinct genetic sources were compared, the seedlings from the most southerly sources exhibited significantly superior height growth than those from more northerly seed sources (Figure 2). Only seedlings from seed orchard SO1-6 did not follow this tendency, which may be due to fewer environmental constraints in the region surrounding SO1-6 compared to those of other northern sources represented by orchards SO1-3, SO1-4, and SO1-5 (Figure 1). The length of the growing season may also partially explain these results, given that southern provenances of white spruce (Li et al., 1993) and other boreal species (Li et al., 1997b; Grossnickle, 2000; Beaulieu et al., 2004) generally exhibit late bud set.

Although seedlings from second-generation seed orchards showed better height growth than those from first-generation seed orchards, their response to variations of climatic conditions on plantation test sites was similar to those of first-generation sources. This trend suggests a similar plasticity to the climatic gradient tested between seedlings from first- and second-generation seed orchards. Many genetic studies involving forest tree species have shown that selective breeding has little or no effect on the degree of adaptation to local conditions (MacLachlan et al., 2017a,b). In the case of white spruce, the species is essentially undomesticated and has a wide genetic diversity (Jaramillo-Correa et al., 2001), which has not been reduced after one generation of selection (Namroud et al., 2012). As a consequence, one would expect a similar response to variations in site conditions, whether seedlings are from natural provenance stands, first-generation orchards or second-generation orchards. Meanwhile, the lack of more southerly plantations in this study, which would be more representative of the climatic conditions at the origin of the plus trees that made up the second-generation orchards (in particular, the trees that were originally from Ontario and southern Québec), limits the ability to verify this hypothesis. In fact, it is possible that the differences in plasticity between the two seed orchard types would be more visible in more southerly site conditions. To verify this, field tests of genetic material from the two generations of seed orchards would need to be established in southern Ontario, for example.

Height growth of seedlings from each of the seed sources was highest on the intermediate site (Asselin) (Figures 2, 4). Better height growth in the central parts of the natural range of black spruce (*Picea mariana* (Mill.) and jack pine (*Pinus banksiana* Lamb.) have been observed previously (Thomson and Parker, 2008; Thomson et al., 2009; Pedlar and McKenney, 2017). These studies suggested that more southerly populations may benefit from a cooler environment that better corresponds to their historical adaptation optimum prior to recent climatic warming (Andalo et al., 2005), and northern populations from a milder climate south of their native origin due to their physiological plasticity. Indeed, average growing season temperatures on the three test sites during the 4-year study were higher than the climatic normal over a 30-year period (1981–2010). Conditions at the intermediate Asselin site (14.6°C) were more in line with the climate normals experienced by southern populations than conditions at the southerly Watford site (15.8°C; Figure 1). The highest average precipitation during the growing season (460 mm), observed at the Watford site, and the small numbers of consecutive days without precipitation (5.8–6.7 days) confirmed that this difference could not be explained alternatively by a period of drought.

Our results show the beneficial use of assisted migration for the southern seed sources, which would have a better performance in actual cooler conditions. Northern sources would benefit from the local warming, but without being able to attain the growth performance of southern sources. Further studies regarding the cold tolerance of southern sources planted on northern sites (e.g., thermal acclimation, cold hardiness, drought tolerance) should be undertaken to provide information on the risks of assisted migration, such as those due to epigenetic effects as illustrated Norway spruce (Skrøppa et al., 2007).

Recently, several studies have highlighted the relative contribution of epigenetics phenomena, which might explain some of the variation in tree performance and other adaptive traits by the maternal environment effect (climatic condition during seed production) (Yakovlev et al., 2012). This phenomenon has been reported to influence bud burst, bud set and cold acclimation in Norway spruce (*Picea abies*) (Johnsen et al., 2005; Skrøppa et al., 2007, 2010), and early growth in maritime pine (*Pinus pinaster*) (Zas et al., 2013). Similar results have been reported for white spruce (Stoehr et al., 1998). However, seeds used in this study were produced under very narrow climatic conditions (2 years), which did not deviate from climate normals for each genetic source. As such, we might conclude that the results reflect local adaptation to climate of seed origin. Nevertheless, more research is needed to disentangle the contribution of epigenetic memory and local adaptation in tree performance and adaptation capacity in order to put in place a more efficient climate change adaptation strategy.

The chipping of tree branches on the southerly Watford site before the seedlings were planted may have caused a temporary immobilization of nitrogen (elevated C/N) (Holland and Coleman, 1987) inducing the reduced growth on this site. While symptoms of nitrogen deficiency were observed in some seedlings during the first growing season, 4 years after planting, the C/N ratio at the Watford site decreased substantially, reflecting good decomposition of organic matter and the availability of nitrogen. In addition, the vector analysis did not show any nitrogen deficiency.

During the 4 years after planting, few changes were observed in the ranking of seed sources for height growth on each of the sites. The significant correlation observed between the initial height at planting (measured under nursery conditions at the end of the 2012 growing season) and height after four growing seasons in plantation (2016) corroborates the results of other studies that showed correlations between nursery and plantation growth of white spruce at different ages (from 4 to 8 years) for families (Li et al., 1993) and clones (Wahid et al., 2013). This relationship has also been documented for black spruce seven to 13 years after outplanting (Williams et al., 1987). This trend indicates that an early selection in the nursery, or at a young age, of the best performing genotypes should be considered to provide improved juvenile growth during the critical phase of seedling establishment on forest sites.

Seed sources presenting the best height growth also exhibited a greater accumulation of biomass, for both roots and above-ground tissue (Figures 2, 3). The proportion of total seedling biomass that was allocated to roots, or the R/S ratio, increased with latitude of test sites, as previously reported for several other forest tree species (Reich et al., 2014). A shorter growing season at the northerly site of Deville may have favored root growth, which is usually most vigorous before and after the growing season (Lahti et al., 2005). However, certain authors have shown that root growth decreases with temperature (Lamhamedi and Bernier, 1994; Grossnickle, 2000; Lahti et al., 2005). The present results are not congruent with this tendency (Figure 2), and suggest that seedlings modified their allocation of biomass in response to harsher environmental conditions. For instance, the northerly Deville site received, on average, less precipitation (333 mm) than the intermediate Asselin site (388 mm) and the more southerly Watford site (460 mm) during the four growing seasons. Moreover, a lower soil temperature, as observed at the more northerly Deville site, would typically result in an increased water viscosity and a reduction in the active absorption of water and nutrients (Grossnickle, 2000). The addition of these factors may emulate the effect of water or nutrient stress, forcing the seedlings to allocate more resources to root growth in an attempt to improve the absorption of available water and mineral elements (Zadworny et al., 2016).

Regarding the presence of adventitious roots, the absence of any significant effect of planting sites, seed sources, and their interaction 4 years after outplanting, indicates a similar expression of this trait among sites and seed sources. However, a pronounced variation was observed within seed sources (from 0 to 21 adventitious roots per plant). This variability may be due, for the most part, to the genotype of the individual and the environmental conditions of the microsite specific to each seedling, notably planting depth (Aubin, 1996), the accumulation of organic matter which may be favored by the micro relief (presence of depressions), soil water content (Aubin, 1996), soil fertility, and the young age of the plantation being studied. With respect to this last factor, the development of adventitious roots was only observed 5–6 years after planting on reforestation sites (Gingras et al., 2002; Parent et al., 2003; Tarroux et al., 2014). In addition, white spruce is characterized by high variation in root growth among clones (Lamhamedi et al., 2000; Wahid et al., 2012, 2013) and in growth and architecture of adventitious roots of cuttings among families (Gravel-Grenier et al., 2011).

### Carbon allocation and nutritional status of seedlings

The absence of interaction between the sites and seed orchards indicates that the seed sources have a similar plasticity for this trait. Seedlings from four seed sources (SO1-2, SO1-3, SO1-6, and SO2-2) displayed the best carbon sequestration, while three sources (SO1-2, SO1-6, and SO2-2) also showed better height growth. Similarly, the best carbon sequestration was observed at the intermediate site, Asselin, which also exhibited the best height growth. Therefore, juvenile growth seems to be a good indicator of juvenile carbon sequestration and could likely be used as a criterion when selecting the best genetic sources for carbon sequestration.

Nitrogen concentrations observed in the above-ground seedling parts at the nursery stage (1.50–1.68%) (Villeneuve et al., 2016) decreased to values between 1.08 and 1.27% at the southerly Watford site, between 1.10 and 1.17% at the intermediate Asselin site and, between 1.02 and 1.32% at Deville, the northerly site. Vector analysis showed that this reduction in concentration was accompanied by an increase in dry mass and nitrogen content, which translated into a general dilution of nitrogen content in the above-ground parts (Figure 6). Successful establishment after outplanting is largely dependent on foliar nitrogen reserves (Grossnickle, 2000). In Québec, a minimum foliar nitrogen concentration of 1.5% is recommended for good seedling growth (Gagnon and Lamhamedi, 2011), but a threshold of 2% should assure optimal performance (Landis, 1989). It is well known that nitrogen is limited in the boreal forest ecozone (Foster and Bhatti, 2006; Lupi et al., 2013) and that during planting and establishment, demand is primarily met by the seedling's reserves (Munson and Bernier, 1993). Furthermore, some authors proposed intensive nitrogen loading as an approach to reach the target concentrations and favor optimal growth after outplanting (Timmer, 1997). This strategy promotes luxury consumption and a strong accumulation of nitrogen in foliar tissues. These nitrogen reserves, during the establishment phase, allow the seedlings to achieve high photosynthetic rates before (Table 3) and after bud break (Gagnon and Lamhamedi, 2011), thus favoring the accumulation of the sugars necessary for bud break, the formation of new shoots (Tranquillini, 1979; Grossnickle, 2000) and root growth (Gagnon and Lamhamedi, 2011). Moreover, it has been shown that good survival and juvenile growth are positively correlated with high foliar nitrogen concentrations (Thiffault and Jobidon, 2006), in addition to better biomass production and nutrient absorption (Timmer and Munson, 1991). Good seedling establishment on the three planting sites and the general nitrogen dilution observed in the present study support these observations and clearly show that foliar nitrogen concentrations of at least 1.5% under nursery conditions favor good seedling establishment after outplanting.

### Photosynthetic traits before bud break

Photosynthesis before bud break allows seedlings to replenish energy reserves depleted by respiration during the winter (Tranquillini, 1979) and accumulate the sugars necessary for bud break and the subsequent elongation of new shoots (Tranquillini, 1979; Grossnickle, 2000). The absence of a significant difference between sites and seed sources demonstrates the lack of local adaptation to climatic conditions for this trait as well as good plasticity for all of the seed sources tested in this study. The present northerly conditions did not appear limiting for photosynthesis before bud break and the more northerly seed sources used in this study did not exhibit a significant advantage under these conditions. In addition, northward migration of southern genetic sources does not appear to incur significant risks for photosynthesis before bud break, within the geographical limits and climatic gradient tested in this study. However, these results are drawn from a study of juvenile seedlings in plantation and can only be confirmed through continued monitoring until the trees reach maturity.

The photosynthetic capacity before bud break was significantly correlated with stomatal conductance, as was similarly demonstrated for photosynthesis during the growing season (Benomar et al., 2015, 2016). Stomatal conductance was lowest in the southern site, which may be due to a lower average soil temperature at this site (5.5°C) when photosynthetic measurements were made. Note that photosynthetic measurements were first made at Watford and subsequently at Asselin and Deville, respectively, with at least 5 days required for each site. This explains why soil temperature was lower at Watford when data were collected and could have led to an increased water viscosity (Grossnickle, 2000) during the sampling period at this site. This state of water reduces its absorption by the roots and negatively affects stomatal conductance (Lamhamedi and Bernier, 1994). Larger root systems with more fine roots, like those observed at the intermediate Asselin and northerly Deville sites, could have also favored better water absorption (Zadworny et al., 2016), partially offsetting this negative effect.

## Conclusions and implications for assisted migration

In this study we evaluated growth, nutritional status and variation in photosynthetic traits before bud break of seedlings of eight distinct white spruce seed sources 4 years after outplanting. Overall, this study revealed that the eight seed orchards have similar plasticity as indicated by the lack of interaction between sites and seed orchards. Photosynthetic-related traits before budbreak and root morphology were similar among seed orchards and were not correlated with height growth. Mineral deficiencies were not observed for any seed orchard at any site, suggesting that the high accumulation of foliar nitrogen in the nursery (>1.5%) favored good seedling establishment.

These results obtained for young seedlings after four growing seasons indicate that the southern seed sources may already benefit from a migration to cooler conditions. Otherwise, a decrease in growth, stemming from partial maladaptation to warmer local conditions, could be observed. On the other hand, the northern seed sources could probably benefit from the predicted warmer regime, due to their physiological plasticity, but their growth performance would likely never attain that of southern sources moved to more northern locations, because they are better genetically adapted to a warmer climate. Studying a larger latitudinal gradient would also be beneficial and could further highlight the differences in local genetic adaptation of white spruce seed sources, while helping to better detect the presence of genotype-environment interactions and identify the functional limits of physiological plasticity.

Because of the positive correlations between height performances in nursery and 4 years after outplanting on forest sites, early selection of seed sources with the best height growth could be conducted. Moreover, juvenile height growth seems to be a good indicator of the juvenile carbon sequestration and could serve as a selection criterion for the best genetics sources for carbon sequestration. Nonetheless, long-term monitoring remains necessary to confirm that these early results hold over time.

## Author contributions

This paper is part of the M.Sc. thesis of GO who was supervised by JBe and ML. ML, JBe, JBo, AR, and LB conceived the study and obtained the funding. All authors participated in the drafting of the manuscript. AR participated to designing the genetic tests, providing the seed and establishing and maintaining tests on the field. ML and his team installed the three weather stations for the acquisition of environmental data, and conducted mineral and root architecture analyses. JD participated in the statistical analyses. GO performed traits measurements, data analyses and primary interpretation of results. All authors read and approved the final version of the manuscript.

### Conflict of interest statement

The authors declare that the research was conducted in the absence of any commercial or financial relationships that could be construed as a potential conflict of interest.
