# Supplementary material for: Ecophysiology and Growth of White Spruce Seedlings from Various Seed Sources along a Climatic Gradient Support the Need for Assisted Migration
Source: Front Plant Sci. 2018 Jan 8;8:2214. doi: 10.3389/fpls.2017.02214 (PMC5766665; doi:10.3389/fpls.2017.02214)
Supplement: Supplementary file 1 [file Image1.pdf]

Supplementary materials

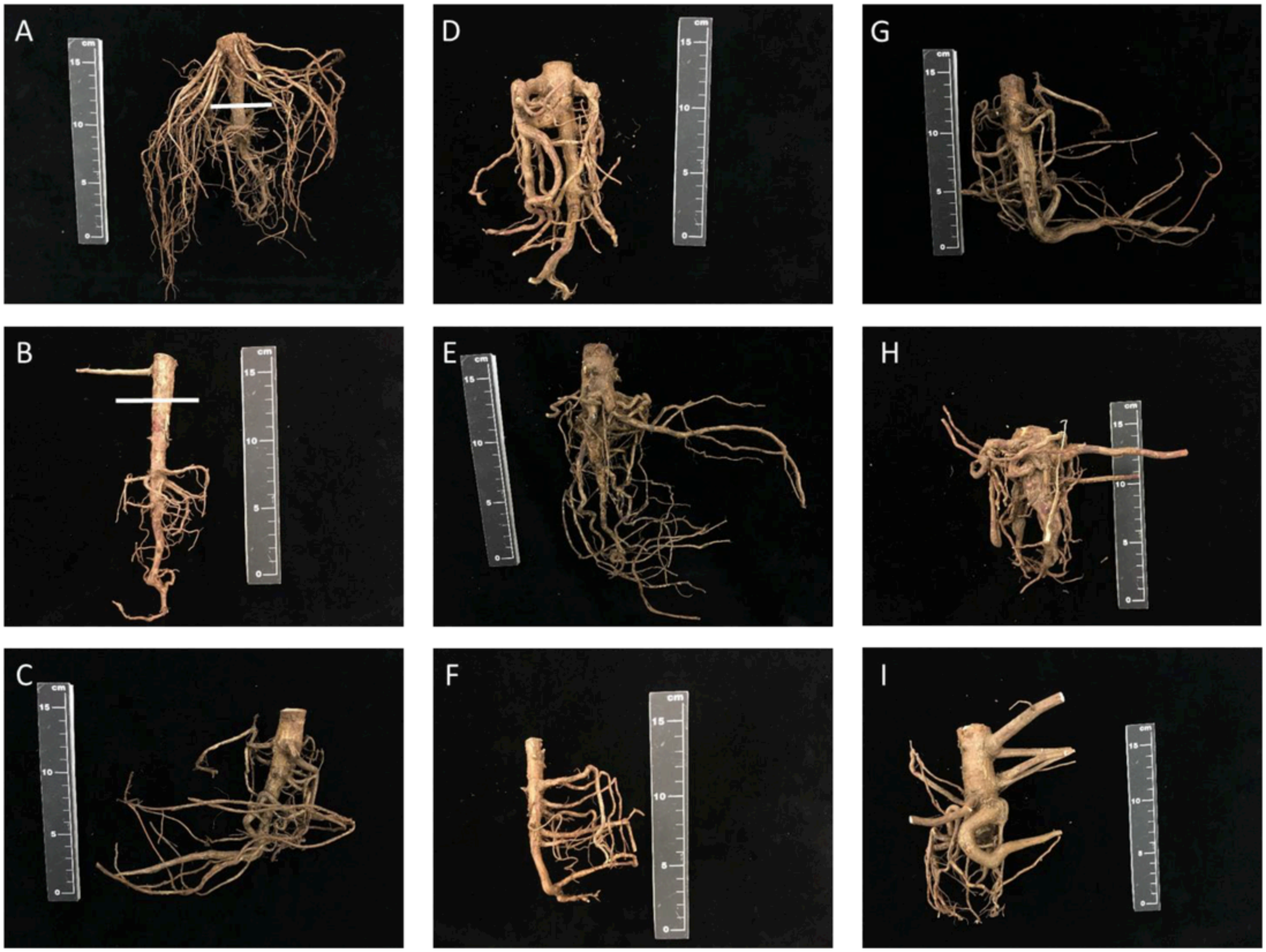

Figure S1. Examples of the observations and classification conducted on the roots, dried and cleaned of fine roots, of seedlings from different seed orchards grown on the three plantation study sites. The first column shows the presence/absence of adventitious roots (adventitious roots are separated from the root mass by a white line); (A) many adventitious roots, (B) one adventitious root and (C) no adventitious roots. Second column shows root orientation; (D) equal distribution on each side, (E) tendency towards one side and (F) only one side. Third column shows examples of root deformation; (H) L- root, (I) swollen root and (J) J-root.

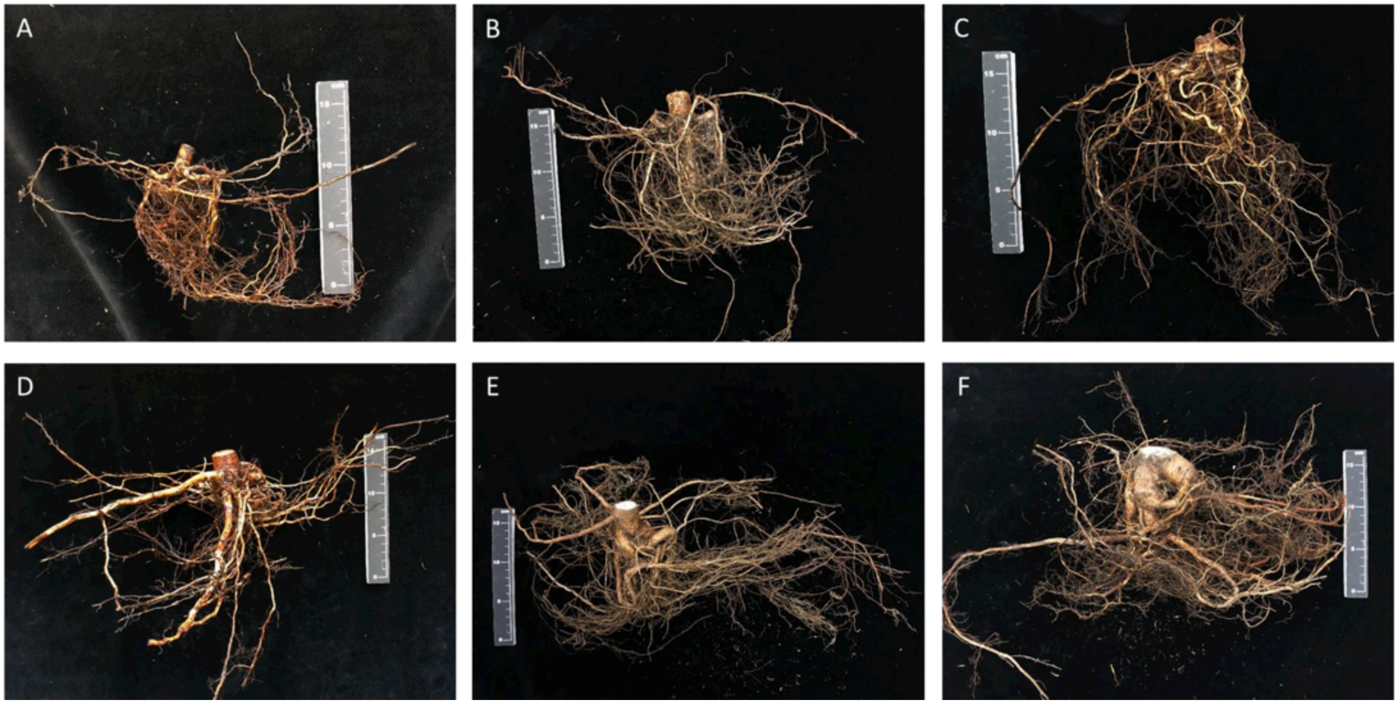

Figure S2. Examples of the differences in complete intact root systems (not dried and with fine roots) as a function of plantation study sites. The columns represent the forest sites of Watford (south), Asselin (intermediate) and Deville (north), respectively, and the rows represent the seed orchards SO1-4 (upper) and SO1-2 (lower).

Table S1: ANOVA table of seed orchard growth traits measured during 4 growing season for height (n=6444) and after the fourth growing season for the others traits (n=96) on three planting sites located along a climatic gradient.

	Site		Seed orchard		Site * Seed orchard		Year		Site * Year		Seed orchard * Year		Site * Seed orchard * Year	
	(df = 2)		(df = 7)		(df = 14)		(df = 3)		(df = 6)		(df = 21)		(df =42)	
	**F**	***P***	**F**	***P***	**F**	***P***	**F**	***P***	**F**	***P***	**F**	***P***	**F**	***P***
Height	184.4	< 0.001	11.1	< 0.001	0.5	0.942	2817.6	< 0.001	201.6	< 0.001	2.0	0.009	1.0	0.474
Adv. Root biomass	1.7	0.297	1.8	0.211	2.2	0.139								
Root biomass	8.3	0.009	3.3	0.005	0.9	0.552								
Shoot biomass	8.6	0.008	2.9	0.010	1.2	0.308								
Needle biomass	5.6	0.027	2.2	0.046	0.8	0.622								
Total biomass	12.5	0.003	3.3	0.005	0.9	0.608								

Adv. root biomass, adventitious root biomass
